# The American Agriculturist

**Published:** 1877-02

**Authors:** 


*The American Agriculturist--One Help for Hard Times.*—To increase the product of one's labor, or business, and then to make the best use of what is obtained, will certainly be helpful in these hard times, or in any other. The hints and suggestions of half a dozen intelligent, practical men and women, who devote themselves to study and observations, on just this topic, must certainly be of great utility to every one. We shall, therefore, do our readers a favor by directing their attention to that most valuable practical journal, the *American Agriculturist*, which is just now entering upon its 36th year. It is packed full of useful information, that cannot fail to be very helpful to every family, and to every man whatever his calling, and whether residing in City, Village, or Country. Each Volume gives from 600 to 700 fine original engravings. that are both pleasing and instructive—to Housekeepers and Children, to Farmers, Mechanics, Merchants, Professional Men, indeed to all classes. Its House Plans and improvements, with full particulars of cost, etc., with engravings, its fearless exposure of humbugs and quackery, indeed its whole make up and its thoroughly reliable character, render it worthy of a place in every household, and we strongly advise every one to have it. An immense circulation enables the Publishers to supply it at the low cost of $1.60 a year, post-paid, or four copies for $5.40. Take our advice and send now for volume 36, to the Publishers, Orange Judd Company, 245 Broadway, New York City.

